# 酸性酯交换-气相色谱-质谱法同时测定植物油中氯丙二醇酯和缩水甘油酯

**DOI:** 10.3724/SP.J.1123.2021.05009

**Published:** 2022-02-08

**Authors:** Xueting WANG, Jingjing LI, Shan JIANG, Weijian SHEN, Yiqian WANG, Qiang GU

**Affiliations:** 1.南京海关动植物与食品检测中心, 江苏 南京 210001; 1. Animal, Plant and Food Inspection Center, Nanjing Customs, Nanjing 210001, China; 2.张家港海关综合技术中心, 江苏 张家港 215600; 2. Comprehensive Technology Center of Zhangjiagang Customs, Zhangjiagang 215600, China

**Keywords:** 酸性酯交换, 气相色谱-质谱法, 氯丙二醇酯, 缩水甘油酯, 植物油, acidic transesterification, gas chromatography-mass spectrometry(GC-MS), monochloropropanediol esters, glycidyl esters, vegetable oils

## Abstract

建立了一种气相色谱-质谱同时测定植物油中3-氯丙二醇酯、2-氯丙二醇酯和缩水甘油酯的方法。称取0.25 g样品,依次加入内标工作液、四氢呋喃和酸性溴化钠溶液,50 ℃水浴反应15 min,加入6 g/L碳酸氢钠溶液终止反应,使用正己烷提取,上层液经氮气吹干后用四氢呋喃溶解。随后加入1.8%(v/v)硫酸-甲醇溶液于40 ℃恒温水浴中反应16 h,加入饱和碳酸氢钠溶液终止反应。样液再经过净化、衍生、提取、氮吹后,以1 mL正己烷定容,过膜,进样测定。采用毛细管气相色谱柱DB-5MS(30 m×0.25 mm×1 μm)分离,程序升温,电子轰击电离(EI)源检测,在选择离子扫描模式下,以保留时间和特征离子信息进行定性分析,内标法定量。结果表明,3-氯丙二醇酯、2-氯丙二醇酯和缩水甘油酯在0.01~0.80 mg/L范围内线性关系良好,相关系数(*r*^2^)均在0.999以上,方法的检出限(*S/N*=3)和定量限(*S/N*=10)分别为25、25、20 μg/kg和75、75、60 μg/kg。选取4种不同基质类型的样品,在低、中、高3个不同添加水平下的平均回收率为89.0%~98.7%,相对标准偏差(RSD)在2.05%~7.81%之间。采用该方法测定了市售112份植物油样本,其中有84份样本检出3-氯丙二醇酯、2-氯丙二醇酯和缩水甘油酯。与已建立的国家标准方法(GB 5009.191-2016)和行业标准方法(SN/T 5220-2019)相比,该方法所采用的酸性酯交换法可避免副反应(碱性条件下3-氯丙二醇、2-氯丙二醇及3-溴丙二醇向游离态缩水甘油转化)的发生,同时该方法也弥补了国家标准和行业标准无法对3-氯丙二醇酯、2-氯丙二醇酯和缩水甘油酯同时进行测定的缺失。该方法实验操作更高效,结果更准确、重复性更好,对我国植物油中3-氯丙二醇酯、2-氯丙二醇酯和缩水甘油酯污染水平的控制、检测标准的制定和生产工艺的优化具有一定的理论和现实意义。

## Abstract (en)

A comprehensive analytical method based on gas chromatography-mass spectrometry (GC-MS) was developed for the determination of 3-monochloropropanediol esters, 2-monochloropropanediol esters, and glycidyl esters in vegetable oils. Different parameters, such as bromination reaction temperature, bromination reaction time, derivatization reagent dosage, and derivative reaction time, were studied. The optimal conditions were as follows: 0.25 g of oil was weighed in a 10-mL glass tube, followed by the addition of 2 mL tetrahydrofuran, 25 μL of internal working standard solutions, and 30 μL of acid aqueous solution of NaBr, homogenized, and the mixture was incubated at 50 ℃ for 15 min. The reaction was stopped by the addition of 3 mL of an aqueous solution of sodium hydrogen carbonate. To separate the oil from the water phase, *n*-heptane was added, and the upper layer was transferred to an empty test tube and evaporated to dryness under a nitrogen stream. The residue was dissolved in 1 mL of tetrahydrofuran. 1.8 mL of sulfuric acid solution in methanol was added to the sample, and the resulting mixture was incubated at 40 ℃ for 16 h. The reaction was stopped by the addition of 0.5 mL of an aqueous solution of sodium hydrogen carbonate. After purification by *n*-hexane and derivatization of phenylboric acid, the derivatives were extracted with *n*-hexane. After nitrogen blowing, the residue was dissolved in 1 mL of *n*-hexane, and then filtered through a 0.45-μm membrane filter unit prior to GC-MS analysis. Temperature programming was applied at an initial temperature of 80 ℃. After 0.5 min, the temperature was raised to 180 ℃ at a rate of 20 ℃/min, held for 0.5 min, raised to 200 ℃ at a rate of 5 ℃/min for 4 min, and finally raised to 300 ℃ at a rate of 40 ℃/min for 4 min. The target compounds were separated on a DB-5MS column (30 m×0.25 mm×1 μm). Identification and quantification were achieved using an electron impact (EI) ion source in the positive ion mode with the selected ion monitoring mode. The internal standard method was used to quantify the 3-chloropropanediol esters, 2-chloropropanediol esters, and glycidyl esters. Under the optimal conditions, the correlation coefficients of the standard calibration curves were greater than 0.999 in the mass concentration range of 0.01-0.80 mg/L. The limits of detection were 25, 25, and 20 μg/kg (*S/N*=3), and the limits of quantification were 75, 75, 60 μg/kg (*S/N*=10). Four samples of different matrix types were selected for scaling experiments. At spiked levels of 250, 500, and 750 μg/kg, the recoveries of 3-chloropropanediol esters, 2-chloropropanediol esters, and glycidyl esters in spiked samples ranged from 89.0% to 98.7%, with relative standard deviations between 2.05% and 7.81% (*n*=6). This method was used to determine 112 commercially available vegetable oil samples, among which 84 samples were detected with 3-chloropropanediol esters, 2-chloropropanediol esters, or glycidyl esters.

The method developed in this study was remarkably different from the standard method, which are mentioned in the national standard method (GB 5009.191-2016) and industry standard method (SN/T 5220-2019), especially in the pretreatment step that involved acidic transesterification. Use of the acidic transesterification method can avoid side reactions, such as the conversion of 3-chloropropanediol, 2-chloropropanediol, and 3-bromopropanediol to free glycidol under alkaline conditions. The method developed in this study was more efficient, and the results were more accurate and reproducible. It has theoretical and practical significance for the control of 3-chloropropanediol esters, 2-chloropropanediol esters, and glycidyl esters residues in vegetable oils, establishment of detection standards, and optimization of the production process.

氯丙二醇酯(monochloropropanediol esters, MCPDEs)是氯丙二醇与脂肪酸的酯化产物,其中3-氯丙二醇酯(3-monochlorpropanediol esters, 3-MCPDEs)在胰酯酶作用下会释放出具有潜在致癌性的3-氯丙二醇(3-MCPD)^[1]^。有文献报道,在未精炼的原油中几乎不含有3-MCPDEs(<0.05 mg/kg),然而在精炼油中3-MCPDEs的含量却会显著增加(0.2~20 mg/kg)^[2]^。缩水甘油酯(glycidyl esters, GEs)是缩水甘油与脂肪酸的酯化产物,是制备3-MCPDEs的前体物,本身并不具有致癌性。研究表明,精炼油中10%~60%的3-MCPDEs是由GEs转化而来的^[3]^。现如今3-MCPDEs和GEs已成为继反式脂肪酸之后油脂精炼加工过程中产生的新型潜在危害因子^[4]^。到目前为止,我国尚未设立油脂中MCPDEs和GEs的限量值,但2018年欧盟发布的(EU)2018/290号法规已对油脂中GEs总量给出了界限值。为严格控制加工油脂中MCPDEs和GEs的含量水平,确保食用植物油的安全,建立可靠的分析方法是首要工作。

现阶段国内外已建立了多种分析方法测定3-MCPDEs^[5,6,7,8,9,10]^,但2-氯丙二醇酯(2-monochlorpropanediol esters, 2-MCPDEs)和GEs的定量分析方法发展相对滞后。已报道的GEs检测方法有液相色谱-质谱联用法^[11,12]^(直接法)和气相色谱-质谱联用法^[13,14,15,16,17,18,19]^(间接法),其中间接法只需一个单一的酯类标准品就可测得GEs总量因而更适合于常规分析。在使用间接法测定MCPDEs和GEs的研究中,根据酯交换反应使用的催化剂类型不同,间接法又分为碱催化间接法和酸催化间接法。碱催化间接法由于酯交换反应时间快,检验周期短,已有很多文献报道,国家标准和海关总署发布的行业标准也都采用了此法,但是在碱性介质中3-MCPD、2-氯丙二醇(2-MCPD)和3-溴丙二醇(3-MBPD)很容易发生副反应生成游离态的缩水甘油,进而会造成过低评估样品中3-MCPDEs、2-MCPDEs和GEs总量,影响检测结果的准确性^[20,21,22]^。此外,国家标准和行业标准无法实现3类物质的同时测定;行业标准检测最终结果将GEs的总量以3-MCPD计也与欧盟限量要求不符。基于上述原因,本方法以美国油脂化学协会AOCS Cd 29a-13方法^[23]^为基础,增加了2-MCPDEs的同位素内标,选取了响应高且无干扰的监测离子,并通过优化前处理条件建立了一种酸性酯交换间接分析法,它可以同时对植物油中3-MCPDEs、2-MCPDEs和GEs的总量进行准确定量分析,且定量方式完全符合欧盟要求。在试样制备过程中,我们使用酸性酯交换反应有效避免3-MCPD、2-MCPD和3-MBPD副反应的发生;通过对溴化反应温度、溴化反应时间、衍生化试剂用量等前处理条件的优化,取得较好的效果。该方法重现性好,准确度高,适用于实验室对植物油中这3类加工污染物的常规检测。

## 1 实验部分

### 1.1 仪器、试剂与材料

7890B-5977A气相色谱-质谱联用仪(美国Agilent科技有限公司); SQP Quintix224-1CN电子分析天平(赛多利斯科学仪器(北京)有限公司); KH-500SP超声波振荡器(昆山禾创超声仪器有限公司); BS-06恒温水浴摇床(韩国jeiotech公司); WH-3涡旋混合器(上海沪西分析仪器厂); Auto Vap S60氮吹仪(美国ATR公司)。

正己烷、丙酮、甲醇、四氢呋喃、甲苯均为色谱纯(美国Fisher公司);溴化钠、碳酸氢钠、硫酸钠、硫酸均为优级纯(国药集团);苯基硼酸(phenyl boronic acid, PBA,纯度97%,美国Sigma公司);实验用水为GB/T 6682规定的一级水。

3-氯-1,2-丙二醇棕榈酸双酯(纯度99.6%,英国LGC公司), D_5_-3-氯-1,2-丙二醇棕榈酸双酯(纯度98%,加拿大TRC公司), 2-氯-1,3-丙二醇硬脂酸双酯(纯度98%,加拿大TRC公司), D_5_-2-氯-1,3-丙二醇硬脂酸双酯(纯度98.2%, BePure公司),棕榈酸缩水甘油酯(纯度98.0%, BePure公司), D_5_-棕榈酸缩水甘油酯(纯度96%,加拿大TRC公司)。

112批油脂样品来源于本地超市及电商销售平台。

### 1.2 实验方法

1.2.1 标准溶液的配制

分别准确称取3-氯-1,2-丙二醇棕榈酸双酯标准品53.14 mg、D_5_-3-氯-1,2-丙二醇棕榈酸双酯标准品51.27 mg、2-氯-1,3-丙二醇硬脂酸双酯标准品58.21 mg、D_5_-2-氯-1,3-丙二醇硬脂酸双酯标准品56.13 mg、棕榈酸缩水甘油酯标准品42.18 mg、D_5_-棕榈酸缩水甘油酯标准品40.15 mg至6个10 mL棕色容量瓶中,以甲苯为溶剂配制质量浓度均为1000 mg/L的单标准储备液(以对应的氯丙二醇、氘代氯丙二醇、缩水甘油和氘代缩水甘油计), -20 ℃避光保存,有效期2年。

将氯丙二醇酯和缩水甘油酯单标准储备液(1000 mg/L)用甲苯稀释配制成10 mg/L(以对应的氯丙二醇和缩水甘油计)的混合标准工作液,-20 ℃避光保存,有效期1年。

将内标储备液(1000 mg/L)用甲苯稀释配制成10 mg/L(以对应的氘代氯丙二醇和氘代缩水甘油计)的混合内标工作液,-20 ℃避光保存,有效期1年。

1.2.2 样品前处理

准确称取0.25 g(精确至0.0001 g)植物油样品于10 mL螺口玻璃试管中,准确加入25 μL混合内标工作液,再加入2 mL四氢呋喃,涡旋溶解,作为待反应液。

向待反应液中加入30 μL酸性溴化钠溶液(溴化钠3 mg/mL,含5%(v/v)硫酸),旋紧盖子,涡旋混合,在50 ℃水浴反应15 min;加入3 mL碳酸氢钠溶液(6 g/L)终止反应。随后加入2 mL正己烷,涡旋振荡15 s,静置,取上清液于另一10 mL螺口玻璃试管中,再用正己烷重复提取1次,合并提取液,40 ℃氮吹至近干,加入1 mL四氢呋喃溶解残渣,作为待净化液。

向待净化液中加入1.8 mL硫酸-甲醇溶液(1.8%, v/v),涡旋混合10 s,旋紧瓶盖,置于40 ℃恒温水浴中反应16 h。加入0.5 mL饱和碳酸氢钠溶液终止反应,涡旋10 s,氮吹除去有机溶剂。再向剩余液中依次加入2 mL硫酸钠溶液(200 g/L), 2 mL正己烷,涡旋混合10 s,静置分层后,弃去上层正己烷,再用正己烷重复萃取1次,弃去上层正己烷,得到下层净化液备用。

向净化液中加入300 μL饱和苯基硼酸溶液,涡旋10 s后,于室温下超声5 min。取出后,加入2 mL正己烷,涡旋10 s,静置分层后,取上层正己烷至另一洁净的玻璃试管中。再用正己烷重复提取1次,合并提取液,40 ℃氮吹至近干。用1 mL正己烷溶解残渣,涡旋10 s,经0.45 μm尼龙微孔滤膜过滤后进行测定。

1.2.3 分析条件

GC条件 DB-5MS毛细管柱(30 m×0.25 mm×1 μm);进样口温度280 ℃;升温程序为80 ℃保持0.5 min,以20 ℃/min速率升温至180 ℃,保持0.5 min,再以5 ℃/min速率升温至200 ℃,保持4 min,最后以40 ℃/min速率升温至300 ℃,并保持4 min;进样方式为脉冲不分流;进样量1 μL。

MS条件 电子轰击离子源(EI);电离能量70 eV;离子源温度280 ℃;四极杆温度150 ℃,传输线温度280 ℃;扫描方式为选择离子扫描(SIM);溶剂延迟6 min;定时事件14 min MS关闭;3种目标化合物及其内标的保留时间和定量、定性离子见表1。

**表 1:** 3种目标化合物及其内标的保留时间和定量、定性离子

Target compound	Retention time/min	Quantitative ion (m/z)	Qualitative ions (m/z)
3-MCPD-PBA	10.960	147	91, 196
D_5_-3-MCPD-PBA	10.898	203	93, 150
2-MCPD-PBA	11.561	198	104, 196
D_5_-2-MCPD-PBA	11.488	201	93, 104
3-MBPD-PBA	12.885	240	91, 146
D_5_-3-MBPD-PBA	12.795	245	149, 150

3-MCPD-PBA: 3-monchloropropane-1,2-diol-phenylboronic derivative; D5-3-MCPD-PBA: d5-3-monchloropropane-1,2-diol-phenylboronic derivative; 2-MCPD-PBA: 2-monchloropropane-1,3-diol-phenylboronic derivative; D5-2-MCPD-PBA: d5-2-monchloropropane-1,3-diol-phenylboronic derivative; 3-MBPD-PBA: 3-monobromopropane-1,2-diol-phenylboronic derivative; D5-3-MBPD-PBA: d5-3-monobromopropane-1,2-diol-phenylboronic derivative.

## 2 结果与讨论

### 2.1 3种目标化合物仪器条件的建立

本方法的建立是基于在酸性条件下,GEs的环氧环易受亲核试剂的攻击而被打开,生成单酰基甘油样分子。由于溴化物与氯化物的相似性较高,在质子溶剂中反应活性较大,当亲核试剂是含溴离子的卤化物时,GEs会转化为2-MBPD单酯或3-MBPD单酯。已有研究表明,GEs上脂肪酸链引起的空间位阻效应使其以形成3-MBPD单酯为主(3-MBPD与2-MBPD的信号响应比在13~15之间)^[23]^。因2-MCPD、3-MCPD和3-MBPD具有相似的分子结构,我们可以使用同一种衍生试剂——苯基硼酸,实现对样品中2-MCPDEs、3-MCPDEs和GEs的同步定量分析。

将3-氯-1,2-丙二醇棕榈酸双酯、2-氯-1,3-丙二醇硬脂酸双酯及棕榈酸缩水甘油酯用甲苯配制成10 mg/L的单标准溶液,D_5_-3-氯-1,2-丙二醇棕榈酸双酯,D_5_-2-氯-1,3-丙二醇硬脂酸双酯及D_5_-棕榈酸缩水甘油酯用甲苯配制成5 mg/L的单标准溶液,经卤化、水解、衍生后,用全扫描方式进行测定,以此获得各目标化合物的特征离子。3种目标化合物及其内标在GC-MS分析条件下,经全扫描模式分析得到相应的总离子流图和质谱图见图1。

**图1:**
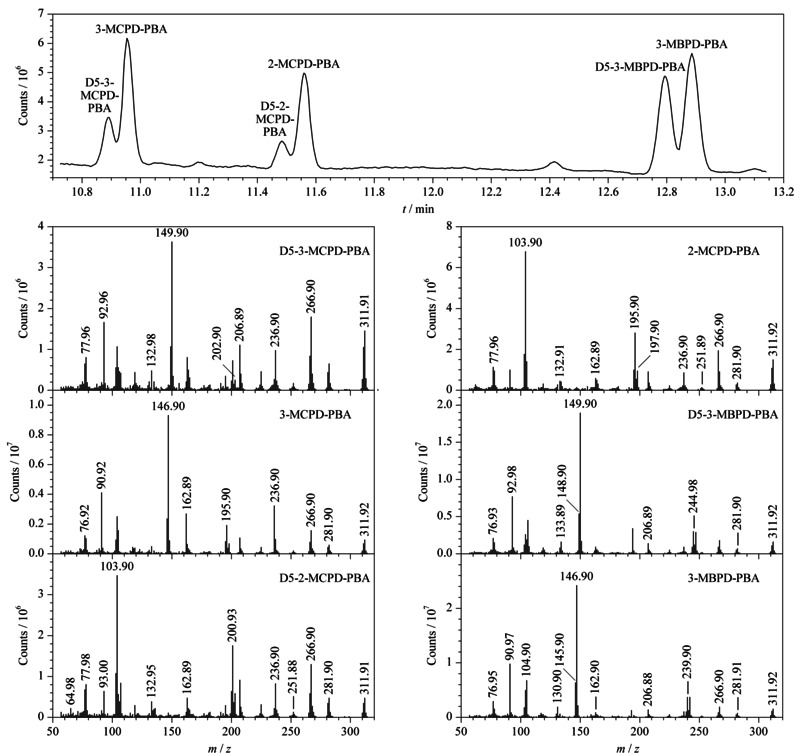
3种目标化合物及其内标的总离子流图和质谱图

从图1中可以看出,3种目标化合物在该GC-MS分析条件下响应及分离度良好;选取响应高且无干扰的监测离子,可以降低噪声和其他干扰效应,提高检测灵敏度,在选择离子监测模式下,3种目标化合物及其内标的定量和定性离子见表1。

### 2.2 前处理条件的优化

2.2.1 溴化反应条件的优化

Hrncirik等^[24]^已详细研究了硫酸和亲核试剂溴化钠浓度对缩水甘油酯转化成3-MBPD单酯效率的影响,本方法直接引用其结果,仅考察了溴化反应温度(30、40、50、60和70 ℃)和溴化反应时间(5、10、15、20和25 min)对缩水甘油酯转化成3-MBPD单酯效率的影响。准备待测样品10份,在样品中加入100 μL混合内标工作液,在不同的溴化反应温度和溴化反应时间下,按照1.2.2节进行处理,内标D_5_-3-MBPD-PBA的峰面积结果见图2。

**图2:**
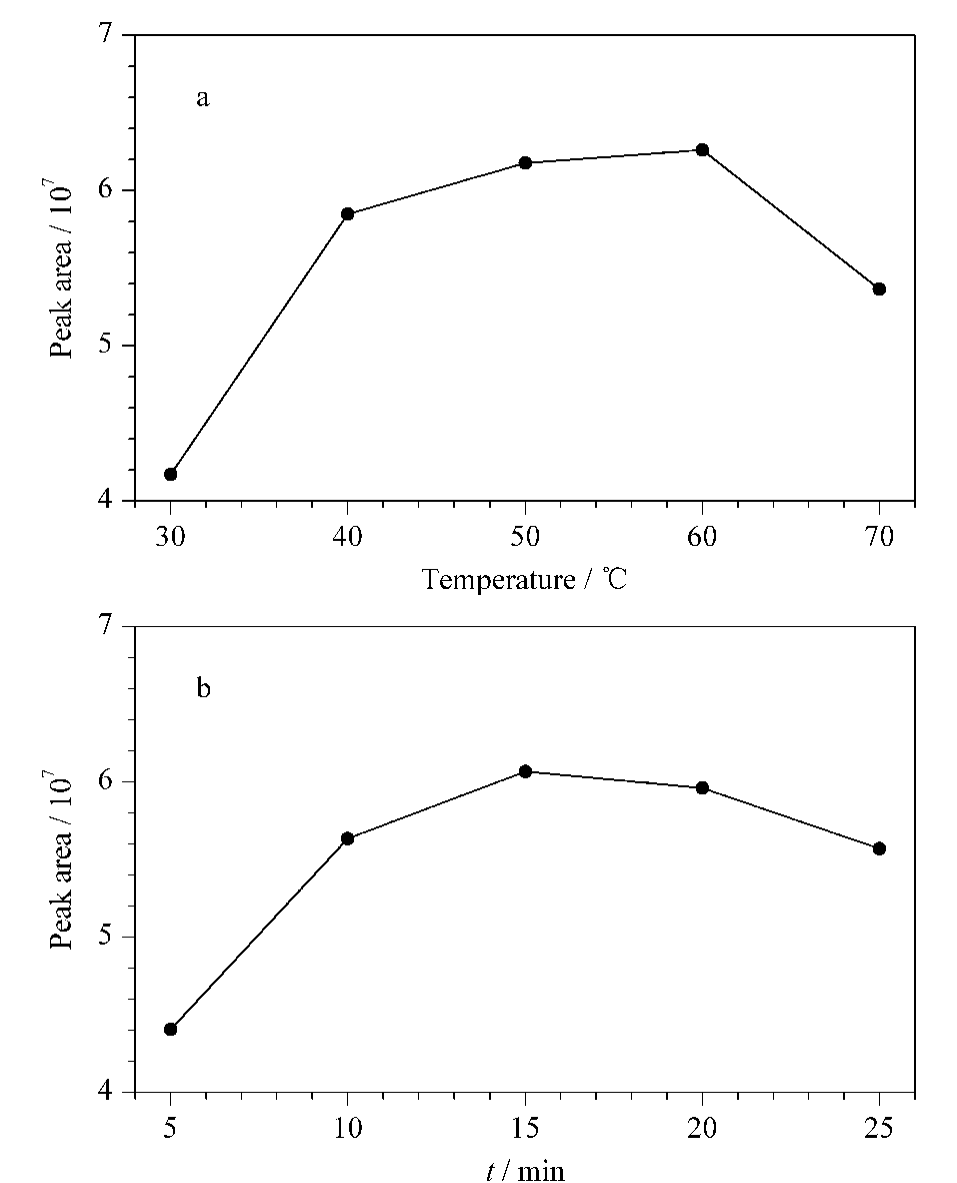
(a)溴化反应温度和(b)溴化反应时间对植物油样品中 内标D_5_-棕榈酸缩水甘油酯转化效率的影响

从图2中可以发现,较高的温度和较长的反应时间都会对缩水甘油酯的转化效率造成影响。为获得缩水甘油酯的最佳转化效率,最终我们选取50 ℃下反应15 min作为溴化反应的最佳条件。

2.2.2 衍生化反应条件的优化

3-MBPDEs、3-MCPDEs、2-MCPDEs及其同位素内标物经硫酸-甲醇水解后分别生成了3-MBPD、D_5_-3-MBPD、3-MCPD、D_5_-3-MCPD、2-MCPD和D_5_-2-MCPD,再经PBA衍生后得到3种目标化合物及3种内标化合物,即3-MBPD-PBA、D_5_-3-MBPD-PBA、3-MCPD-PBA、D_5_-3-MCPD-PBA、2-MCPD-PBA和D_5_-2-MCPD-PBA。考察了衍生化试剂的使用体积(100、200、300、400和500 μL)和衍生化反应时间(5、10、15、20和25 min)对测定结果的影响。

准备待测样品10份,将样品中加入500 μL混合内标工作液,在衍生化试剂不同使用量和反应时间下,按照1.2.2节进行处理,3种内标化合物的峰面积结果见图3。

**图3:**
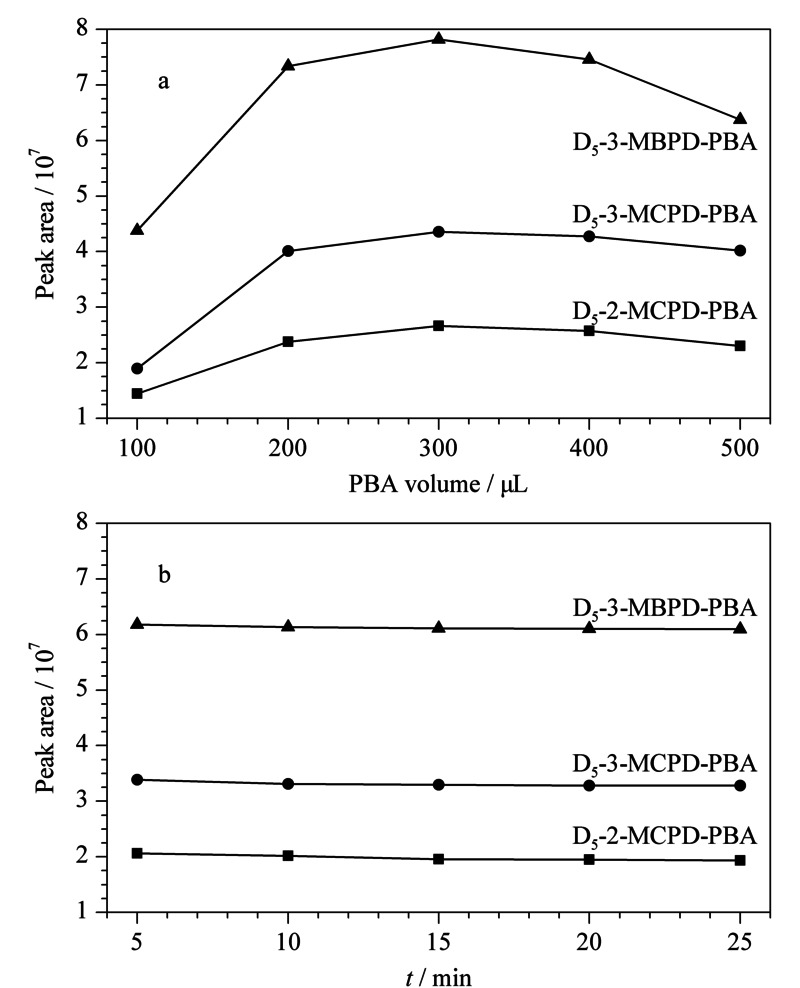
(a)衍生化试剂的使用体积和(b)衍生化反应时间对植物油样品中3种内标测定结果的影响

如图3a所示,衍生化试剂的用量对内标D_5_-棕榈酸缩水甘油酯的影响最为明显,综合考虑3种内标的信号响应情况,最终我们选取衍生化试剂用量为300 μL;图3b显示,衍生化反应时间对3种内标的信号响应值影响较小,在5 min时衍生化反应已基本完成,出于节约时间方面考虑,最终选择衍生化时间5 min。

### 2.3 方法学指标考察结果

2.3.1 标准曲线、检出限和定量限

准确移取混合标准工作液和内标工作溶液适量于6个10 mL螺口玻璃试管中,按1.2.2节步骤与试样同时处理。得到质量浓度为0.01、0.05、0.10、0.20、0.40和0.80 mg/L的系列混合标准溶液,其中内标的质量浓度均为0.25 mg/L,按浓度由低到高的顺序进样分析。

以目标化合物及其对应氘代同位素内标的浓度比值为横坐标,以目标化合物及其对应氘代同位素内标的峰面积比值为纵坐标,绘制标准曲线,从而获得线性方程和相关系数(*r*^2^),见表2。结果表明,线性相关系数均大于0.999,说明目标化合物在0.01~0.80 mg/L范围内具有良好的线性关系。

以定量离子信噪比(*S/N*)为3和10时的响应定义方法的检出限(LOD)和定量限(LOQ), 3-MCPDEs、2-MCPDEs及GEs的检出限和定量限分别为25、25、20 μg/kg和75、75、60 μg/kg。

**表 2:** 目标化合物的线性方程、相关系数、检出限和定量限

Compound	Linear equation	*r* ^2^	LOD/ (μg/kg)	LOQ/ (μg/kg)
3-MCPD-PBA	*y*=17.770068*x*-0.196156	0.9998	25	75
2-MCPD-PBA	*y*=0.391145*x*+0.001431	0.9998	25	75
3-MBPD-PBA	*y*=0.722427*x*-0.007760	0.9999	20	60

*y*: peak area ratio of the analyte to the internal standard; *x*: mass concentration ratio of the analyte to the internal standard.

2.3.2 准确度和精密度

按照前述方法,对大豆油、菜籽油、葵花籽油和亚麻籽油4种样品进行添加回收试验,设定添加水平为250、500、750 μg/kg,考察方法的准确度和精密度,结果见表3。不同基质中3-MCPDEs、2-MCPDEs和GEs的加标回收率为89.0%~98.7%,相对标准偏差(RSD)为2.05%~7.81%,实验结果表明该方法具有良好的准确性和精密度。

**表3:** 3-MCPDEs、2-MCPDEs和GEs在植物油样品中的添加回收率和相对标准偏差(*n*=6)

Oil type	Spiked/ (μg/kg)	3-MCPDEs				2-MCPDEs				GEs		
		Measured/ (mg/kg)	Recovery/ %	RSD/%		Measured/ (mg/kg)	Recovery/ %	RSD/ %		Measured/ (mg/kg)	Recovery/ %	RSD/ %
Soybean oil	0	ND	-	-		ND	-	-		ND	-	-
	250	0.244±0.015	89.3	5.98		0.258±0.012	89.0	4.60		0.274±0.014	90.3	4.94
	500	0.478±0.019	91.8	3.98		0.505±0.017	94.3	3.33		0.523±0.019	95.2	3.72
	750	0.736±0.018	95.9	2.44		0.740±0.018	94.5	2.37		0.758±0.017	95.2	2.26
Rapeseed oil	0	0.216±0.014	-	6.37		0.117±0.009	-	7.81		0.409±0.017	-	4.21
	250	0.439±0.018	90.0	4.02		0.351±0.015	94.4	4.16		0.644±0.020	94.6	3.15
	500	0.677±0.025	92.8	3.71		0.578±0.019	92.9	3.31		0.869±0.025	92.6	2.92
	750	0.931±0.027	96.1	2.91		0.840±0.020	97.2	2.39		1.144±0.028	98.7	2.43
Sunflower oil	0	ND	-	-		ND	-	-		0.107±0.003	-	2.93
	250	0.240±0.010	91.7	4.21		0.258±0.010	90.9	3.91		0.335±0.013	91.9	3.79
	500	0.476±0.019	93.5	4.08		0.487±0.019	91.5	3.86		0.573±0.019	93.9	3.23
	750	0.725±0.016	96.1	2.26		0.764±0.017	98.7	2.17		0.809±0.025	94.8	3.15
Linseed oil	0	ND	-	-		ND	-	-		ND	-	-
	250	0.243±0.013	91.3	5.49		0.247±0.010	91.0	4.22		0.256±0.013	91.3	5.22
	500	0.483±0.021	94.0	4.27		0.488±0.018	94.0	3.76		0.493±0.019	93.5	3.93
	750	0.744±0.019	97.6	2.53		0.736±0.015	95.9	2.05		0.740±0.017	95.4	2.35

ND: not detected.

### 2.4 本方法与标准方法测定值的比较

采用本文所建立的方法、国家标准方法(GB 5009.191-2016)和行业标准方法(SN/T 5220-2019)测定了植物油(样品编号:FAPAS 2662)中3-MCPDEs、2-MCPDEs和GEs的值,结果见表4。从表4可知,由于标准方法中使用了甲醇钠-甲醇溶液进行酯键断裂反应,易造成3-MCPD、2-MCPD和3-MBPD副反应的发生,实验结果的重复性不好且测定值会偏低一些。

**表4:** 不同方法测得的植物油中3种目标物的含量(*n*=6)

Analyte	Assigned value/ (mg/kg)	Contents/(mg/kg)		
		This method	GB 5009.191-2016	SN/T 5220-2019
3-MCPDEs	0.569	0.558±0.016	0.498±0.033	0.516±0.031
2-MCPDEs	0.275	0.265±0.012	0.211±0.024	-
GEs	0.459	0.456±0.015	-	-

### 2.5 市售样品分析评价

采用本文建立的方法对市售的112批植物油,包括大豆油、菜籽油、葵花籽油、玉米油、芝麻油样品进行分析,在84批样品中检出3-MCPDEs、2-MCPDEs和GEs(测定值范围见表5),检出率高达75%;部分大豆油、菜籽油和芝麻油中GEs含量高出欧盟(EU)2018/290号法规中的限量值(GEs≤1 mg/kg),超标率分别为9.1%、7.5%和45.5%。

**表 5:** 实际样品中3-MCPDEs、2-MCPDEs和GEs的检测结果

Oil type	Sample number	Contents/(mg/kg)		
		3-MCPDEs	2-MCPDEs	GEs
Soybean oil	22	ND-0.970	ND-0.456	ND-1.600
Rapeseed oil	40	ND-1.292	ND-0.549	ND-2.177
Sunflower oil	16	ND-0.398	ND-0.219	ND-0.505
Corn oil	12	0.068-0.509	0.089-0.498	0.108-0.639
Sesame oil	22	0.223-3.670	0.109-1.550	0.059-1.720

## 3 结论

本文所建立的酸性酯交换法既可避免副反应的发生,又可弥补现有标准中无法对3-MCPDEs、2-MCPDEs和GEs同时进行测定的缺失。方法分析效率高、准确度好、重复性佳,对我国植物油中3-MCPDEs、2-MCPDEs和GEs污染水平的控制、检测标准的制定和生产工艺的优化具有一定的理论和现实意义。
